# Hepatoprotective effect of curcumin and alpha-tocopherol against cisplatin-induced oxidative stress

**DOI:** 10.1186/1472-6882-14-111

**Published:** 2014-03-28

**Authors:** Sarawoot Palipoch, Chuchard Punsawad, Phanit Koomhin, Prasit Suwannalert

**Affiliations:** 1 School of Medicine, Walailak University, Nakhon Si Thammarat 80161, Thailand; 2 Department of Pathobiology, Faculty of Science, Mahidol University, Bangkok 10400, Thailand

**Keywords:** Cisplatin, Oxidative stress, Curcumin, α-Tocopherol, Hepatotoxicity

## Abstract

**Background:**

*cis*-Diammineplatinum (II) dichloride (cisplatin) is the important anti-cancer agent useful in treatment of various cancers. Unfortunately, it can produce unwanted side effects in various tissues, including the liver. The present study investigated the possible protective role of curcumin and α-tocopherol against oxidative stress-induced hepatotoxicity in rats upon cisplatin treatment.

**Methods:**

Male Wistar rats were divided into five groups (n = 5). Saline and Cis groups, rats were intraperitoneal (i.p.) injected with normal saline and cisplatin [20 mg/kg body weight (b.w.)], respectively. Cis + α-tocopherol group, Cis + Cur group and Cis + α-tocopherol + Cur group, rats were pre-treated with a single dose of α-tocopherol (250 mg/kg b.w.), curcumin (200 mg/kg b.w.) and combined α-tocopherol with curcumin, respectively, for 24 h prior the administration of cisplatin. After 72 h of first injection, specimens were collected. Liver enzyme, lipid peroxidation biomarker, liver histopathology and gene expression of liver nicotinamide adenine dinucleotide phosphate (NADPH) oxidase were investigated.

**Results:**

Cisplatin revealed a significant increase of hepatic malondialdehyde (MDA) levels and a significant reduction of hepatic superoxide dismutase (SOD) and catalase activities compared to the saline group. It elicited a marked increase of the serum alanine aminotransferase (ALT) and aspartate aminotransferase (AST) levels and demonstrated the liver pathologies including liver congestion, disorganization of hepatic cords and ground glass appearance of hepatocytes. It also demonstrated a significant increase of NADPH oxidase gene expression compared to saline group. Pre-treatment with combined curcumin and α-tocopherol improved the liver enzymes, lipid peroxidation biomarker, liver histopathology and gene expression of liver NADPH oxidase in cisplatin-treated rats.

**Conclusions:**

The findings indicate that pre-treatment with combined curcumin and α-tocopherol can protect cisplatin-induced hepatotoxicity including the biochemical, histological and molecular aspects. The down-regulations of NADPH oxidase gene expression may be involved in abrogating oxidative stress via reduction of reactive oxygen species (ROS) production.

## Background

*cis*-Diammineplatinum (II) dichloride (cisplatin) is the important chemotherapeutic agent useful in the treatment of various cancers [1]. Unfortunately, it can produce side effects in various tissues, including the liver. Although the mechanism of cisplatin-induced adverse effect is still unclear, however several evidences have shown that its hepatotoxicity is believed via reactive oxygen species (ROS) generation-mediated oxidative stress dependent mechanism [2-4]. Oxidative stress is caused by an imbalance between oxidant and antioxidant. It has been implicated in the pathogenesis of various diseases [5,6]. ROS are the highly reactive molecules which are mainly composed of superoxide radical (O_2_^•-^), hydroxyl radical (^•^OH) and hydrogen peroxide (H_2_O_2_). It can damage biological molecules including lipid, protein and DNA and eventually cause disruption of the cellular structural integrity and capacity [7]. NADPH oxidase is a membrane-bound enzyme which has the ability to generate high levels of O_2_^•-^ in response to stimulus [8]. Previous studies revealed that the suppression of NADPH oxidase is able to prevent oxidative stress-induced pathology [9,10].

To prevent oxidative stress, there are several molecules that play a role to scavenge ROS called antioxidant which derived from both exogenous and endogenous sources. Superoxide dismutase (SOD) is one of the most powerful endogenous enzymatic antioxidants which has the ability to convert O_2_^•-^ into H_2_O_2_. The absence of SOD demonstrates the elevated oxidative damage causing various pathologies *in vivo*[11,12]. Catalase is also the important endogenous enzymatic antioxidant responsible for protection of the cell from oxidative damage induced by ROS via converting H_2_O_2_ into water and oxygen. Catalase deficiency leads to oxidative stress by increasing lipid peroxidation as demonstrated in mice [13].

Medicinal plants are good sources of exogenous antioxidants which might be considered as the new alternative approach to ameliorate pathological alterations in oxidative stress-related pathology. Curcumin is derived from the rhizomes of *Curcuma longa* which has been demonstrated to possess antioxidant activity *in vivo*[14]. It has been demonstrated to prevent several pathologies related to oxidative damage such as the inorganic arsenic-induced hepatotoxicity [15]. We hypothesized that treatment with combined curcumin and α-tocopherol may have higher antioxidant ability than treatment with curcumin or α-tocopherol alone. Additionally, α-tocopherol is one of the most biologically active forms of vitamin E. The study aimed at gaining insight into the understanding of the biochemical, histological and molecular effects of curcumin and/or α-tocopherol to protect against cisplatin-induced oxidative stress in rat liver.

## Methods

### Chemicals

*cis*-Diammineplatinum (II) dichloride (product number: 479306, purity ≥ 99.9%), curcumin from *Curcuma longa* (product number: c1386, purity ≥ 65%), α-tocopherol (product number: 258024, purity ≥ 95.5%), Bradford assay kits and 10% neutral buffered formalin solution were purchased from Sigma-Aldrich Chemical Company (St. Louis, MO, USA). Thiobarbituric Acid Reactive Substances (TBARS), SOD and catalase assay kits were obtained from Cell Biolabs, Inc (San Diego, CA, USA). RNeasy mini kit, Omniscript RT kit and HotStar Taq DNA polymerase were obtained from Qiagen (Hilden, Germany). All other chemicals were of analytical grade.

### Animals

Twenty-five male Wistar rats (*Rattus norvegicus*), ranking from 180-200 g, were obtained from the Division of Animal House, Faculty of Science, Prince of Songkla University, Thailand. All animal procedures were reviewed and approved by the Animal Ethics Committee, Walailak University (Protocol number: 004/2012) and were conducted according to the Guide for the Care and Use of Laboratory Animals, National Research Council. Rats were maintained in stainless-steel cages under constant conditions of temperature (23 ± 2°C), relative humidity (50-60%) and lighting (12 h light/dark cycles). Animals were provided with a standard commercial rat diet and distilled water. Animals were acclimatized and closely monitored under laboratory conditions for 2 weeks before the commencement of the experiment.

### Experimental design and specimen collection

Wistar rats were divided into five groups (5 rats per group). Saline group, rats were treated with a single intraperitoneal (i.p.) injection of 1 ml of normal saline. Cis group, rats were injected with a single dose of cisplatin (20 mg/kg b.w.) via i.p. route. Cis + α-tocopherol group, rats were treated with a single dose of α-tocopherol (250 mg/kg b.w.); Cis + Cur group, rats were treated with a single dose of curcumin (200 mg/kg b.w.); and Cis + α-tocopherol + Cur group, rats were treated with a single dose of combined α-tocopherol (250 mg/kg b.w.) and curcumin (200 mg/kg b.w.), via i.p. route for 24 h prior a single dose injection of cisplatin (20 mg/kg b.w.) [16]. Following 72 h of first injection, rats were anesthetized with thiopental sodium intraperitoneally (50 mg/kg b.w.). The peripheral blood from heart was collected in clot activator tubes. Then, rats were euthanized by anesthetizing thiopental sodium overdose (100 mg/kg b.w.). After opening the abdominal cavity, the liver was harvested and immediately washed in ice-cold isotonic saline.

### Determination of serum AST and ALT levels

Blood samples were centrifuged at 3000 rpm for 5 min. Sera were collected and the levels of serum ALT and AST were measured using a Cobas Mira Plus CC Chemistry Analyzer (Switzerland).

### Determination of liver MDA levels

The liver tissue was homogenized to give a final concentration of 50 mg/mL in phosphate buffered saline (PBS) containing 1X butylated hydroxytoluene (BHT), homogenized on ice and centrifuged at 10000 × g for 5 min to collect supernatant. In accordance with the protocol of an OxiSelect^TM^ TBARS Assay Kit (Cell Biolabs, Cat No.: STA-330, San Diego, CA, USA), 100 μL of samples or MDA standard was added to separate microcentrifuge tubes, and then 100 μL of the SDS lysis solution was added and mixed thoroughly. The samples were then incubated for 5 min at room temperature, 250 μL of thiobarbituric acid (TBA) reagent was added, and then each tube was closed and incubated at 95°C for 60 min. The tubes were then removed and cooled to room temperature in an ice bath for 5 min. All sample tubes were then centrifuged at 3000 rpm for 15 min, the supernatant was removed from the samples, and then finally 200 μL of samples and MDA standard was transferred to a 96-well microplate compatible with a spectrophotometric plate reader. The absorbance was read at 532 nm.

### Determination of liver SOD activity

The liver tissue was homogenized to give a final concentration of 50 mg/ml in cold 1X lysis buffer (containing 10 mM Tris, pH 7.5, 150 mM NaCl and 0.1 mM EDTA), centrifuged at 12000 × g for 10 min and the supernatant was collected for analysis. In accordance with the protocol of an OxiSelect™ SOD Activity Assay Kit (Cell Biolabs, Cat No.: STA-340, San Diego, CA, USA), 20 μL of samples, 5 μL of xanthine solution, 5 μL of chromagen solution, 5 μL of 10X SOD assay buffer and 50 μL of deionized water were added (total volume of 90 μL) to a 96-well microplate, and then 10 μL of pre-diluted 1X xanthine oxidase solution was added to each well. The samples were then mixed well and incubated for 1 h at 37°C. The absorbance was read using the spectrophotometric microplate reader at 490 nm.

### Determination of liver catalase activity

The liver tissue was homogenized to give a final concentration of 50 mg/ml in cold PBS with 1mM EDTA, centrifuged at 10000 × g for 15 min at 4°C and the supernatant was collected. In accordance with the protocol of an OxiSelect^TM^ Catalase Activity Assay Kit (Cell Biolabs, Cat No: STA-341, San Diego, CA, USA), 20 μl of the diluted catalase standard or sample and 50 μl of the H_2_O_2_ working solution (12 mM) were added to a 96 well microplate. The samples were then mixed well and incubated for 1 min. The reaction was stopped by adding 50 μl of the catalase quencher into each well and mixed thoroughly, 5 μl of each reaction well was transferred to a fresh well, 250 μl of the chromogenic working solution was added to each well. The samples were then mixed well and incubated for 60 min. The absorbance was read using the spectrophotometric microplate reader at 520 nm.

### Protein determination

Protein content was estimated by Bradford assay (Sigma, USA) using bovine serum albumin (BSA) as standard.

### Liver histology

The liver tissue was preserved in 10% neutral buffered formalin solution for 24 h and washed with 70% ethanol. Tissue was then placed in small metal caskets, stirred by a magnetic stirrer, dehydrated using alcohol series from 70% to 100% alcohol and embedded in paraffin using an embedding machine. Paraffin block was sectioned using a rotary ultra-microtome, distributed onto glass slides and then dried overnight. Slide was observed under a light microscope after being stained with hematoxylin and eosin (H&E) dyes and mounted.

### Liver NADPH oxidase gene expression by reverse transcription-polymerase chain reaction (RT-PCR)

Total RNA was extracted from the liver tissue by RNeasy mini kit. RNA content and purity were measured by a UV spectrophotometer. RT-PCR was done using the extracted RNA for detection of NADPH oxidase gene. For amplification of the targets gene, reverse transcription and PCR were run in two separate steps. Briefly, Reaction mixture of RT reaction containing 1 μg total RNA, 0.5 μg random primer, 5 × RT buffer, 2.5 mmol/l dNTP, 20 U RNase inhibitor and 200 U MMLV reverse transcriptase in a total volume of 25 μl was incubated at 37°C for 60 min, then heated to 95°C for 5 min to inactivate MMLV. PCR was carried out with 1.5 μl RT products, 10 × PCR buffer (without Mg^2+^) 2.5 μl, 2.0 μl dNTP (2.5 mmol/l), 2.0 μl MgCl_2_ (25 mmol/l), 0.5 μl each primer (20 μmol/l) of β-actin, 0.5 μl each primer of gene to be tested (20 μmol/l) and 1 U of Taq DNA polymerase, in a final volume of 25 μl. Thermal cycler conditions were as follows: a first denaturing cycle at 97°C for 5 min, followed by a variable number of cycles of amplification defined by denaturation at 96°C for 1.5 min, annealing for 1.5 min and extension at 72°C for 3 min. A final extension cycle of 72°C for 15 min was included [17]. The primers including:

NADPH oxidase: Forward primer: 5’-GGAAATAGAAAGTTGACTGGCCC -3’

Reverse primer: 5’-GTATGAGTGCCATCCAGAGCAG-3’

Beta actin: Forward primer: 5’TGTTGTCCCTGTATGCCTCT-3’

Reverse primer: 5’-TAATGTCACGCACGATTTCC-3’

The sample was equally loaded on 2% gel agarose, stained with ethidium bromide and visualized by UV transilluminator. The amount of PCR product was quantified using the gel and image analysis software (Syngene, USA).

### Statistical analysis

Results were expressed as mean ± standard error of the mean (S.E.M.). Differences between groups were determined by one-way analysis of variance (ANOVA). Post hoc testing was performed for group comparisons using the Least Significant Difference (LSD) test and *p* < 0.05 was considered significant.

## Results

### Effect of the treatment on liver enzymes

**Figure 1:**
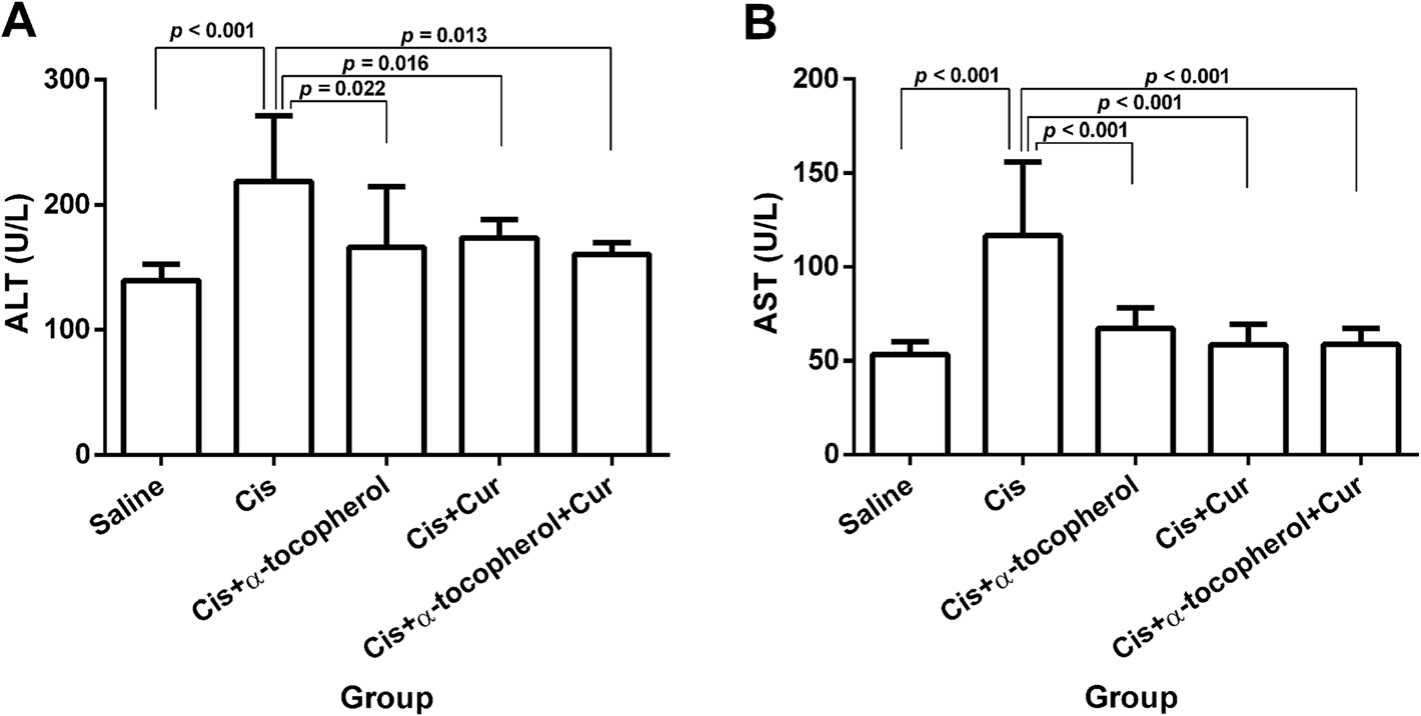
**Serum AST levels (A) and serum ALT levels (B).** Values represent the mean ± S.E.M. (n = 5). Cis: cisplatin, Cur: curcumin.

Cisplatin elicited a significant increase of the serum AST and ALT levels compared to the control (saline) group (*p* < 0.001) (Figures 1A and B). Curcumin and/or α-tocopherol significantly demonstrated the reduction of serum AST levels (*p* < 0.001) (Figure 1A). The alpha-tocopherol group, curcumin group and combined curcumin with α-tocopherol group also significantly elicited the reduction of the serum ALT levels compared to the cisplatin-treated group (*p* = 0.022, *p* = 0.016 and *p* = 0.013, respectively) (Figure 1B).

### Effect of the treatment on lipid peroxidation

**Figure 2:**
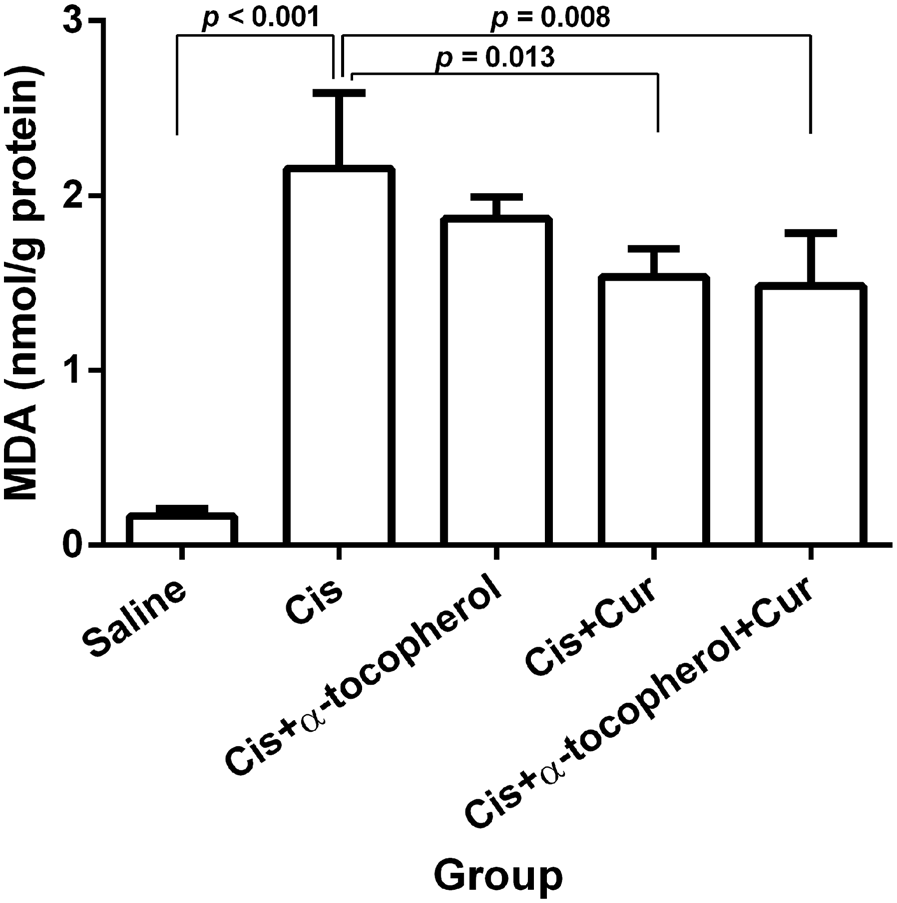
**Liver MDA levels of study groups.** Values represent the mean ± S.E.M. (n = 5). Cis: cisplatin, Cur: curcumin.

Cisplatin demonstrated a marked increase of hepatic MDA levels compared to the saline group (*p* < 0.001) indicating the enhancement of lipid peroxidation (Figure 2). The cisplatin pre-treated with curcumin group and combined curcumin and α-tocopherol group revealed a significant reduce MDA levels compared to the cisplatin-treated group (*p* = 0.013 and *p* = 0.008, respectively).

### Effect of the treatment on enzymatic antioxidant activities

**Figure 3:**
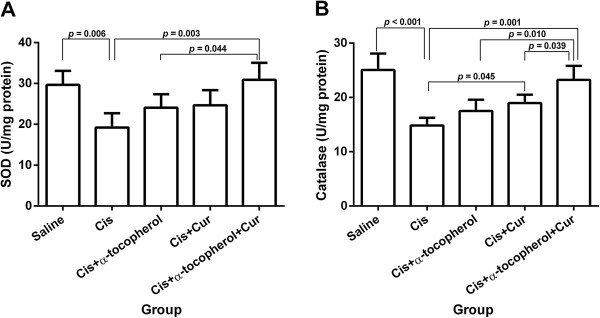
**Enzymatic antioxidant activities of study groups: (A) SOD activity and (B) catalase activity.** Values represent the mean ± S.E.M. (n = 5). Cis: cisplatin, Cur: curcumin.

As shown in Figures 2A and 3B, cisplatin significantly led to the reduction of SOD (*p* = 0.006) and calalase activities (*p* < 0.001) compared to the saline group. The cisplatin pre-treated with combined curcumin and α-tocopherol group significantly elicited the increased SOD activity compared to the cisplatin-treated group (*p* = 0.003) and cisplatin pre-treated with α-tocopherol group (*p* = 0.044) (Figure 3A). The cisplatin pre-treated with curcumin group showed the significant increase of catalase activity compared to the cisplatin-treated group (*p* = 0.045). Moreover, the cisplatin pre-treated with combined curcumin and α-tocopherol group significantly elicited the increase of catalase activity compared to the cisplatin-treated group (*p* = 0.001), cisplatin pre-treated with α-tocopherol group (*p* = 0.010) and cisplatin pre-treated with curcumin group (*p* = 0.039) (Figure 3B).

### Effect of the treatment on liver histology

**Figure 4:**
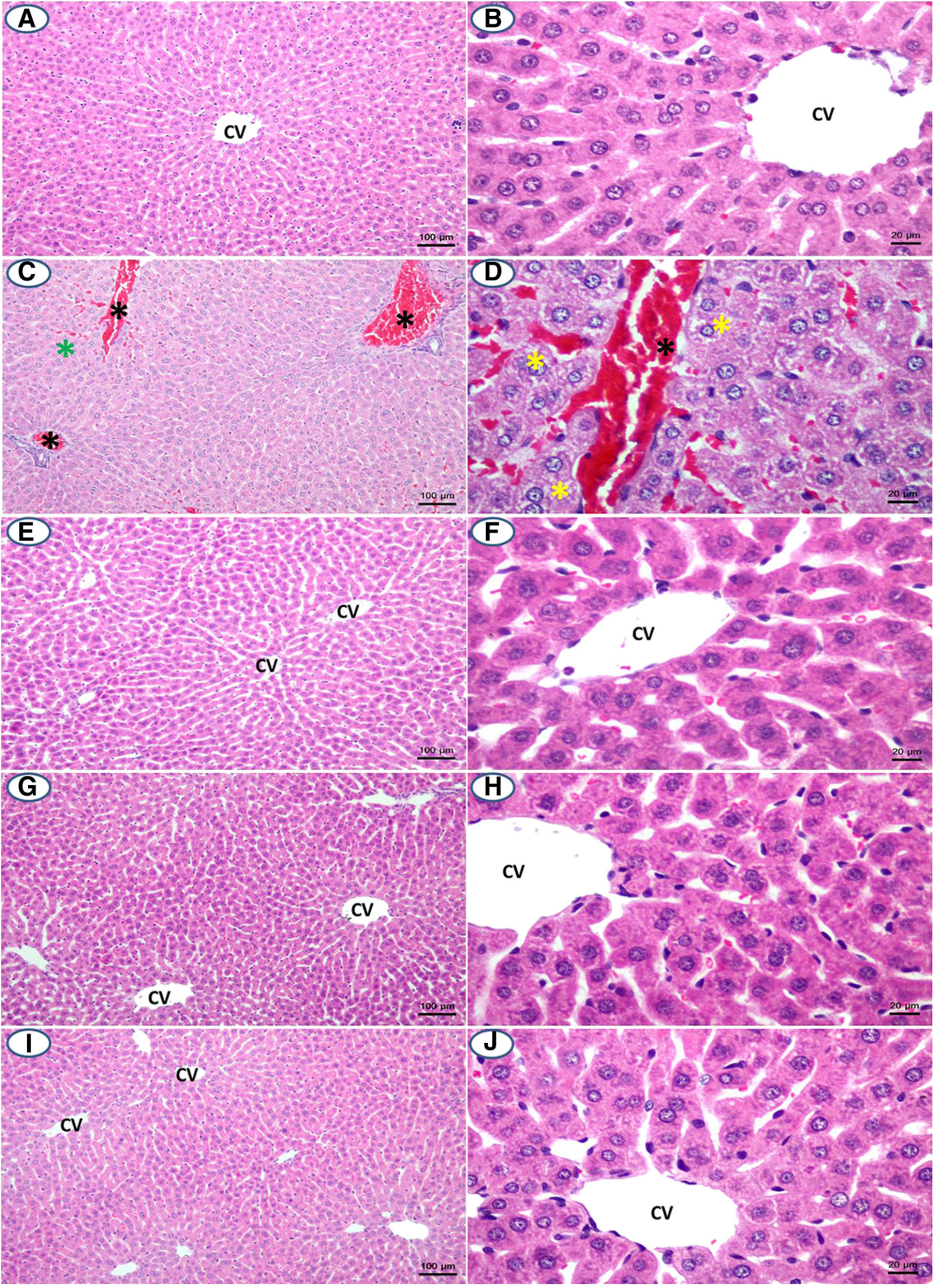
**Liver histology of study groups (H&E staining): (A, B) control group, (C, D) cisplatin-treated group, (E, F) cisplatin pre-treated with α-tocopherol group, (G, H) cisplatin pre-treated with curcumin group and (I, J) cisplatin pre-treated with combined curcumin and α-tocopherol group.** The black asterisk indicates congestion. The green asterisk indicates disorganization of hepatic cords. The yellow asterisk indicates ground glass appearance of hepatocytes. Scale bar = 100 and 20 μm, respectively. CV = central vein.

Cisplatin at 20 mg/kg b.w. caused liver congestion, disorganization of hepatic cords and ground glass appearance of hepatocytes (Figures 4C and D). Pre-treatment with curcumin and/or α-tocopherol was able to improve liver pathology induced by cisplatin at 20 mg/kg b.w. (Figures 4E-J).

### Effect of the treatment on liver NADPH oxidase gene expression

**Figure 5:**
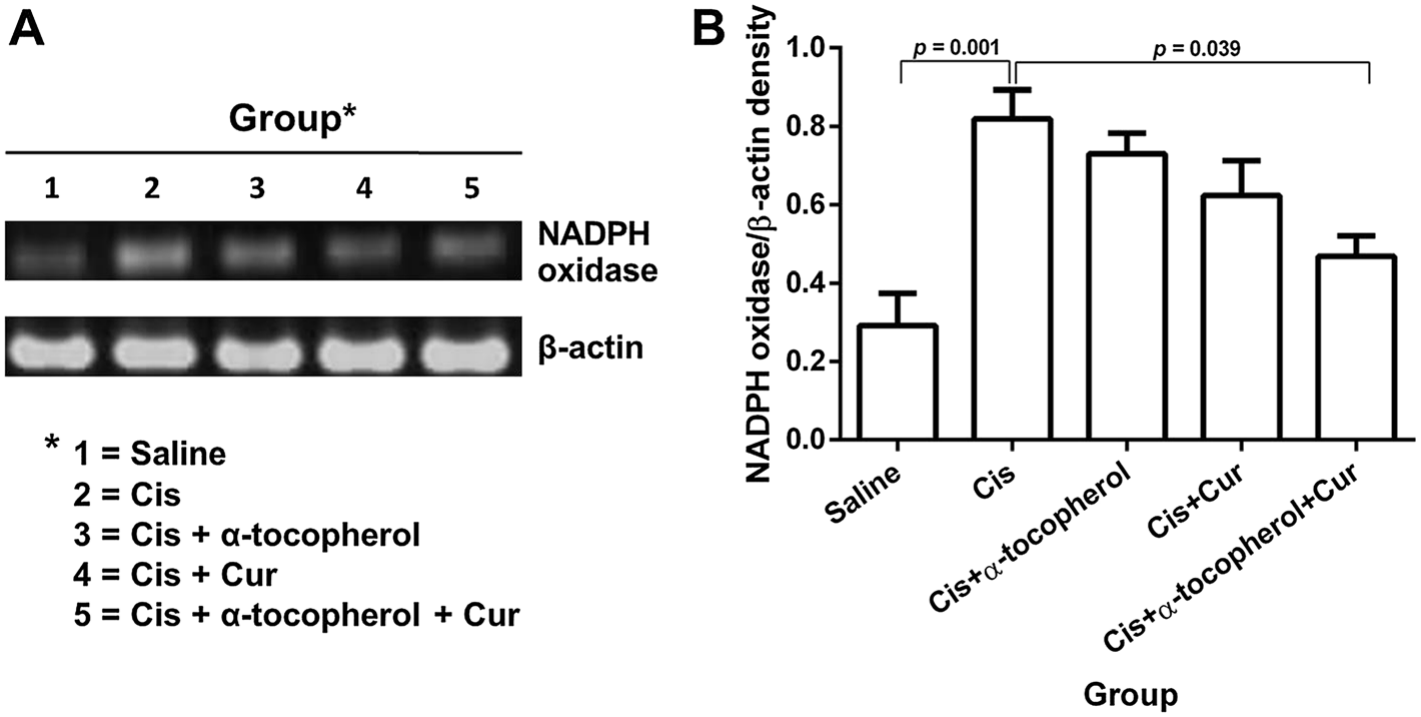
**Liver NADPH oxidase gene expressions in study groups. (A)** agarose gel electrophoresis showing gene expression of NADPH oxidase by RT-PCR and **(B)** density of NADPH oxidase gene compared with β-actin. Values represent the mean ± S.E.M. (n = 5). Cis: cisplatin, Cur: curcumin.

As shown in Figures 5A and B, treatment with cisplatin revealed a significant increase of mRNA levels of NADPH oxidase compared to the saline group (*p* = 0.001). The cisplatin pre-treated with combined curcumin and α-tocopherol-treated groups significantly showed the reduction of mRNA levels of NADPH oxidase compared to the cisplatin-treated group (*p* = 0.039).

## Discussion

In the study, hepatotoxicity induced by cisplatin was recognized by the alterations of the biochemical, histological and molecular parameters. Biochemical alterations included the increase of liver enzyme levels including AST and ALT, the increase of MDA levels and the decrease of SOD and catalase activities. MDA is one of the most potential biomarkers of lipid peroxidation [18]. Histological changes comprised of liver congestion, disorganization of hepatic cords and ground glass appearance of hepatocytes. The molecular alteration indicates the up-regulation of NADPH oxidase gene expression. From this study, we suggest that up-regulation of NADPH oxidase gene expression may be caused the enhancement of ROS production by increasing O_2_^•-^ production, ultimately resulting in generation of lipid peroxidation. Lipid peroxidation is demonstrated to induce disturbance of membrane function and integrity [19]. Moreover, the reduced activities of enzymatic antioxidants led to the decreased scavenging activities against ROS, eventually causing oxidative stress. Reduction of antioxidant defense system was concerned in various pathological conditions induced by oxidative stress. The study indicates that oxidative stress has been implicated in the pathogenesis of cisplatin-induced hepatotoxicity. Many studies have focused on the alternative approach to protect cisplatin-induced hepatotoxicity using natural products-derived antioxidants [2,20,21]. Our work is the first report to demonstrate the combination of curcumin and α-tocopherol for prevention of cisplatin-induced hepatotoxicity.

Curcumin [1,7-bis(4-hydroxy-3-methoxyphenyl)-1,6-heptadiene-3,5-dione] is a natural phenolic compound from turmeric which has demonstrated a variety of pharmacological effects such as antioxidant against hexavalent chromium [Cr(VI)] compound-induced renal oxidant damage, anti-inflammation against neurodegenerative, cardiovascular, pulmonary, metabolic, autoimmune and neoplastic diseases, anti-cancer and enhanced wound healing [22-24]. Curcumin has been reported as the powerful antioxidant in different *in vitro* assays. H-atom donation from phenolic group of curcumin was believed to responsible for the superb antioxidant properties [25]. Moreover, the phenolic compound of curcumin has been confirmed to play the key role in the antioxidant activity [26]. Additionally, curcumin exhibited the highest antioxidant capacity compared to turmeric’s other two curcuminoids including demethoxycurcumin and bisdemethoxycurcumin [27]. This study revealed that pre-treatment with curcumin alone was able to normalize the levels of liver enzymes and lipid peroxidation biomarker, the activity of catalase and liver histopathology in cisplatin-treated rats. The activities of curcumin were similar to previous reports [28,29].

Alpha-tocopherol is an isoform of lipid-soluble vitamin E which acts as a powerful antioxidant [30,31]. The pre-treatment with combined curcumin and α-tocopherol led to the improvement of the liver enzymes levels and lipid peroxidation biomarker, the activities of enzymatic antioxidants, liver histopathology and gene expression of liver NADPH oxidase in cisplatin-treated rats. Previous reports also showed the augmented activity of combined curcumin with other antioxidant compound. Combination of ascorbic acid with curcumin increases the antioxidant activity [32]. Co-administration of vitamin E and curcumin improved the activities of enzymatic antioxidant including cytosolic catalase, cytosolic glutathione peroxidase-1 (GPx1), mitochondrial SOD2 and glutathione reductase, and normalized GPx1 protein expression in l-thyroxine-induced hyperthyroidism in rat [33]. From the results, we suggested that NADPH oxidase play the key role in oxidative stress state induced by cisplatin. Previous studies indicated that NADPH oxidase is the main enzymatic source of ROS production which is responsible for oxidative stress in various diseases via the underlying mechanisms of NADPH oxidase activation [34,35]. This study demonstrated the potent antioxidant property of combined curcumin and α-tocopherol to reduce oxidative damages of liver induced by cisplatin. However, the exact mechanism is still unknown. The down-regulations of NADPH oxidase gene expression may be involved in the abrogation of oxidative stress via reduction of reactive oxygen species (ROS) generation.

## Conclusions

We suggest that oxidative stress has been implicated in the pathogenesis of cisplatin-induced hepatotoxicity by enhancing ROS generation through up-regulation of NADPH oxidase gene and by reducing activities of enzymatic antioxidants. These findings indicate that pre-treatment with combined curcumin and α-tocopherol can protect cisplatin-induced hepatotoxicity including biochemical, histological and molecular aspects. The study provides the evidence of combined curcumin and α-tocopherol as the new adjuvant of cisplatin to abrogate the hepatotoxicity upon cancer chemotherapy.

## Abbreviations

ALT: Alanine aminotransferase; ANOVA: Analysis of variance, AST, Aspartate aminotransferase; b.w: Body weight; BHT: Butylated hydroxytoluene; BSA: Bovine serum albumin; Cisplatin: *cis*-Diammineplatinum (II) dichloride; Cr(VI): hexavalent chromium; DNA: Deoxyribonucleic acid; EDTA: Ethylenediaminetetraacetic acid; GPx: Glutathione peroxidase; H&E: Hematoxylin and eosin; H2O2: Hydrogen peroxide; i.p: Intraperitoneal; LSD: Least significant difference; MDA: Malondialdehyde; MgCl2: Magnesium chloride; NaCl: Sodium chloride; NADPH oxidase: Nicotinamide adenine dinucleotide phosphate oxidase; O2: Superoxide radical; OH: Hydroxyl radical; PBS: Phosphate buffered saline; RNA: Ribonucleic acid; ROS: Reactive oxygen species; RT-PCR: Reverse transcription-polymerase chain reaction; S.E.M.: Standard error of the mean; SDS: Sodium dodecyl sulfate; SOD: Superoxide dismutase; TBA: Thiobarbituric acid; TBARS: Thiobarbituric acid reactive substances.

## Competing interests

The authors declare that they have no competing interests.

## Authors’ contributions

SP conceived this study, and designed the experiments and performed most of the experiments. CP contributed to the liver histology analysis. PK carried out the liver SOD and catalase analysis. All authors analyzed the data, and discussed and concluded the results. SP and PS provided final editing to the manuscript. All authors read and approved the final manuscript.

## Pre-publication history

The pre-publication history for this paper can be accessed here:

http://www.biomedcentral.com/1472-6882/14/111/prepub
